# Human Leg Model Predicts Ankle Muscle-Tendon Morphology, State, Roles and Energetics in Walking

**DOI:** 10.1371/journal.pcbi.1001107

**Published:** 2011-03-17

**Authors:** Pavitra Krishnaswamy, Emery N. Brown, Hugh M. Herr

**Affiliations:** 1 Harvard-MIT Division of Health Sciences and Technology, Massachusetts Institute of Technology, Cambridge, Massachusetts, United States of America; 2 Department of Brain and Cognitive Sciences, Massachusetts Institute of Technology, Cambridge, Massachusetts, United States of America; 3 Department of Anesthesia, Critical Care and Pain Medicine, Massachusetts General Hospital, Harvard Medical School, Boston, Massachusetts, United States of America; 4 The Media Laboratory, Massachusetts Institute of Technology, Cambridge, Massachusetts, United States of America; University College London, United Kingdom

## Abstract

A common feature in biological neuromuscular systems is the redundancy in joint actuation. Understanding how these redundancies are resolved in typical joint movements has been a long-standing problem in biomechanics, neuroscience and prosthetics. Many empirical studies have uncovered neural, mechanical and energetic aspects of how humans resolve these degrees of freedom to actuate leg joints for common tasks like walking. However, a unifying theoretical framework that explains the many independent empirical observations and predicts individual muscle and tendon contributions to joint actuation is yet to be established. Here we develop a computational framework to address how the ankle joint actuation problem is resolved by the neuromuscular system in walking. Our framework is founded upon the proposal that a consideration of both neural control and leg muscle-tendon morphology is critical to obtain predictive, mechanistic insight into individual muscle and tendon contributions to joint actuation. We examine kinetic, kinematic and electromyographic data from healthy walking subjects to find that human leg muscle-tendon morphology and neural activations enable a metabolically optimal realization of biological ankle mechanics in walking. This optimal realization (a) corresponds to independent empirical observations of operation and performance of the soleus and gastrocnemius muscles, (b) gives rise to an efficient load-sharing amongst ankle muscle-tendon units and (c) causes soleus and gastrocnemius muscle fibers to take on distinct mechanical roles of force generation and power production at the end of stance phase in walking. The framework outlined here suggests that the dynamical interplay between leg structure and neural control may be key to the high walking economy of humans, and has implications as a means to obtain insight into empirically inaccessible features of individual muscle and tendons in biomechanical tasks.

## Author Summary

Biological neuromuscular systems are generally able to perform a specified movement task in several ways – as they have significantly more degrees of freedom than mechanical constraints. Understanding how humans resolve these redundancies to drive individual muscles and tendons in typical joint movements is of interest in the fields of biomechanics, neuroscience and prosthetics. Many experimental studies have uncovered neural, mechanical and energetic features of individual muscle and tendon function in common tasks like walking and running. However, a unifying theoretical framework that explains the many independent empirical observations is yet to be established. In this work, we show that leg muscle-tendon morphology and neural co-ordination, together, enable efficient ankle movements in walking. This finding provides quantitative insight into the operation and performance of posterior-leg muscles and tendons in walking, and motivates the idea that different muscle-tendon units take on different mechanical roles to best actuate the ankle in gait. Results reported have implications both for better understanding neuromuscular co-ordination in gait, and for the design of lower limb prosthetic and orthotic technologies.

## Introduction

A common feature in biological neuromuscular systems is the redundancy in joint actuation. Redundancies in actuating a joint with a prescribed force and motion can be classified at three levels. Joints can be actuated by multiple muscle-tendon units (MTUs) working simultaneously. At any instant, energy for MTU work could come from the series elastic tendon or from the active muscle. Each muscle has many sensors and can be controlled by multiple neural pathways acting together. Understanding how these redundancies are resolved in typical joint movements has been a long-standing problem in biomechanics, neuroscience and prosthetics [1], [2].

There is a large literature (reviewed in [3]) on objectives that might drive the way humans resolve neuromechanical redundancies. Several objectives ranging from metabolic cost, efficiency, and mechanical economy to fatigue and active muscle volume have been proposed as driving factors. Direct measurements in humans have revealed some details pertaining to the ‘inner workings’ of individual muscles and tendons resulting from the resolution of neuromechanical redundancies in natural execution of common tasks. Electromyography (EMG) has long quantified neurally stimulated electrical activity (activation) in individual muscles, and indicated which MTUs contribute to joint dynamics during the course of a movement [4]. Recently, ultrasonography has resolved ankle plantar flexor and knee extensor MTU strain into muscle strain and tendon strain during walking, running, and jumping [5]–[7]. Novel approaches using powered exoskeletons to replace leg muscle work have helped estimate the metabolic efficiency of ankle joint actuation in walking [8], [9]. Together, the above studies have uncovered critical neural, mechanical and energetic aspects of individual muscle and tendon contributions to joint actuation.

However, the abundance of research on the (a) driving objectives underlying and (b) empirical observations on redundancy resolution is not accompanied by a unifying theoretical framework that relates the two. There is a need to explain the breakdown of joint actuation, possibly driven by one or more of the above driving objectives, into observed individual element contributions.

Previous studies [10], [11] have proposed that the optimality of neural control for prescribed objectives can resolve individual muscle-tendon contributions to joint actuation in walking. These studies model leg MTUs with morphological parameters based on literature estimates, assert a control objective such as tracking biological joint mechanics or minimizing metabolic cost of transport, and obtain optimal muscle activation profiles for the specified objective. While the importance of neural control in determining the operation of individual muscles and tendons is undisputed, such approaches neglect the fact that many sets of activation patterns can correspond to similar values for the driving objective - making it difficult to uniquely resolve individual muscle activation profiles from an overall mechanical or energetic prescription. Further, several control objectives may be operating in tandem to generate neural activations given the highly non-linear, multi-input multi-output nature of the system - making it difficult to obtain optimal neural activations using a top-down approach. These observations reduce the utility of such approaches for explaining empirical results and making testable predictions on the workings of individual muscles and tendons within the system.

An alternative proposal for resolving individual muscle-tendon contributions to joint actuation in walking is found in optimal design. A starting point for such an approach lies in a recent study by Lichtwark & Wilson [12]. They propose that optimal muscle-tendon design for efficient actuation of an isolated MTU can explain empirically observed muscle and tendon strain profiles within the MTU. The empirically realistic nature of this proposal may directly stem from the well-documented fact that compliant tendons enable muscles to produce force economically [13], [14]. However, this proposal does not scale to explain the breakdown of joint actuation amongst individual elements, as the forces produced by individual MTUs are not known *a priori* for a given joint actuation.

Thus, existing optimal control and optimal design approaches are limited, albeit in different ways, by the very joint actuation redundancies they seek to address. Extra sources of information are needed to address this problem. EMG data contains information about muscle activity, and could potentially be used as a source of biologically realistic neural control commands to muscles. This promises to circumvent the above-mentioned difficulties in obtaining optimal muscle activations. Further, having muscle activation profiles could also enable a more systematic study of the effects of MTU structure (design) on the breakdown of joint actuation amongst individual elements. In other words, estimating muscle activations from the data allows a consideration of both neural control and muscle-tendon design, in tandem, on the operation of individual muscles and tendons.

**Figure 1:**
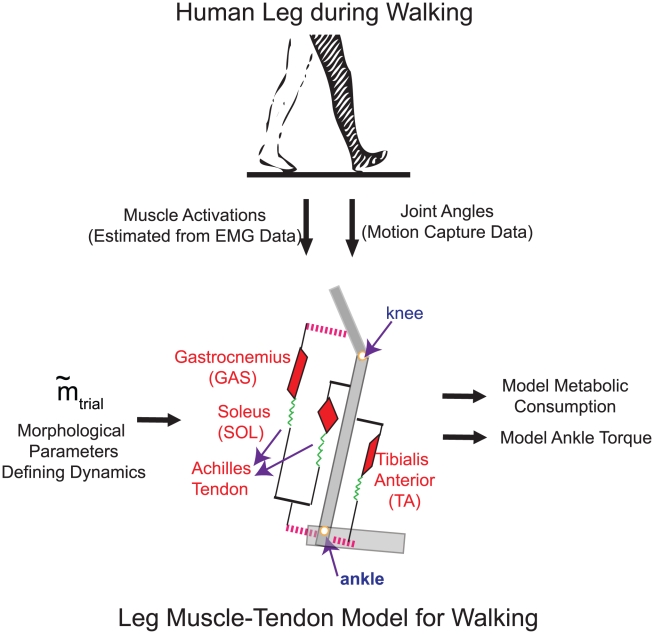
Schematic of model and experiment. The dynamical model of muscle-tendon units contributing to ankle action in walking is shown. Anatomical correlates of the model are indicated. The soleus and gastrocnemius are collectively referred to as plantar flexor muscles. The red triangles denote muscles, green springs denote tendons, the dashed pink lines denote moment arms and gray rectangles denote body segments. The term ‘leg’ is used as per its anatomical definition throughout this paper. All muscle-tendon units are defined with Hill-type dynamics, parameterized with 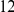 muscle-tendon morphological features 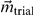 (tendon slack length, tendon material properties and muscle maximum isometric force). For randomly generated parameter vectors 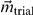, the model was actuated with kinematic and EMG data from healthy subjects. Details on data collection, model dynamics, computation of model torque and metabolic cost are in Methods. The resulting model ankle torque and metabolic consumption were characterized to understand biophysical features underlying the gait data.

Motivated by the above ideas, we have developed a theoretical framework to (a) address how the load of actuating a joint is shared amongst the many MTUs, (b) elucidate features of leg design and neuromuscular control enabling the breakdown and (c) clarify functional advantages arising from the load sharing. As a case study, we examine ankle joint actuation in human walking. We model the three primary leg MTUs contributing to ankle action in walking (Figure 1). Each MTU is characterized by (a) Hill-type muscle dynamics [15], (b) a common non-linear tendon model [16] and (c) a bilinear excitation-activation relation [3] - all of which are assumed to be internally consistent. These relations are parameterized with a minimal set of twelve muscle-tendon morphological features (representing leg MTU design). We conduct a computational exploration of the muscle-tendon design space for correspondence to well-known biological objectives. Specifically, for each set of system parameters, we actuate the model with joint state and muscle activations from healthy human gait data (Methods) to characterize the resulting joint torque and metabolic consumption. An overview of the modeling scheme is presented in Figure 1.

Our results are organized into five sections. First we present our estimates of muscle activations from EMGs recorded during human walking. In the second section, we characterize the leg parameter space by ability to produce human-like ankle torques and economy. We show that there is a unique parameter vector that is able to accomplish both, and that this unique vector corresponds to the maximum metabolic economy. Third, we present the optimal leg parameters, compare them with biological values and discuss their influence on metabolic economy. Fourth, we present model plantar flexor muscle and tendon strain predictions, compare them with two sets of independent empirical recordings and use them to evaluate mechanical power breakdown between muscle and tendon within each MTU. In the fifth section, we present metrics regarding the breakdown of ankle actuation amongst the two different plantar flexors.

## Results

### Estimating Muscle Activation

Muscle activation is an indicator of a muscle's force-generation capability, indicated by the proportion of troponin bound to calcium [17]–[19]. It is driven by neurally stimulated electrical activity in the muscle. Since EMG data is a qualitative indicator of muscle electrical activity [4], it contains valuable information about individual muscle activity and can be useful in understanding the breakdown of joint actuation. However, quantitative uses of EMG data have been limited by variability in the signal and measurement artifacts. Here we show that considering dominant biophysical characteristics of the muscle activation build-up along with the randomness inherent in the EMG measurement yields repeatable and reasonable activation estimates.

Classic EMG analysis involves rectification and low-pass filtering [20], [21]. But low-pass filters smear out the filtered signal, leading to loss of both phase and amplitude information, particularly turn-on and turn-off of muscle activity [22]. Recently Sanger proposed a probabilistic method to resolve the signal variability and noise floor related problems in analyzing EMG signals [22]. In this paper, the muscle electrical activity 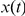 driving the EMG signal was modeled as a jump-diffusion process:
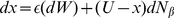
(1)where 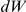 is a diffusion process with rate 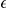, 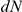 is a jump process with frequency 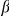 and 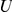 represents a uniform distribution indicating that 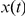 is a uniform random variable when there is a jump. The measured EMG signal was modeled as a random process with an exponential density and rate given by 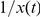:
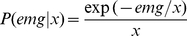
(2)


**Figure 2:**
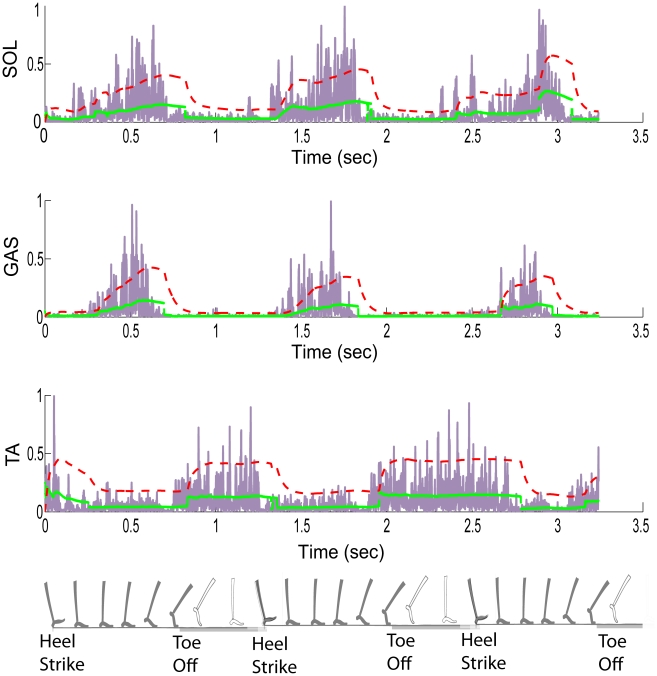
Estimating muscle activations from EMG data. The rectified EMG signal is shown in blue, the Bayesian estimate of muscle electrical activity 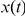 based on [22] is shown in green and activation estimates 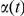 obtained by feeding in the Bayesian estimate through bilinear activation dynamics are in red. The step-like feature of the Bayesian estimate is apparent. The muscle activation estimate builds up after the EMG bursts on, and lasts well after the EMG turns off. Step to step variations in the EMG signals are seen, as are their effects on the activation estimates. The position of the leg corresponding to the time axis is shown for interpretation. The leg is shaded during stance (between heel strike and toe off) and transparent during swing.

Propagating the probability densities in a classic recursive Bayesian manner, to estimate the 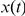 that best describes the observed EMG signal, Sanger reported excellent temporal resolution of EMG turn-on/turn-off during forced maximal contraction tasks. However, the biophysical relevance to analyzing EMG from dynamic tasks is limited by (a) the sharp, near-instantaneous turn-on and turn-off in the Sanger estimates, and (b) the lack of amplitude-buildup when the muscle is on (Figure 2).

We attribute this to differences between the modeled jump-diffusion process and the true buildup of muscle active state in normal tasks (Supplementary Text S2). The constant frequency and uniform amplitude of the jump process [22] compromises the history dependence of active-state buildup, causing sudden jumps in the estimates when the EMG signal turns on/off. Further, the Sanger model has the same jump rate for source and sink or for activation and deactivation. This neglects the differences in activation and deactivation time constants that are critical to muscle activation build-up [19]. Thus the Bayesian approach proposed in [22] appears to estimate the times when muscle electrical activity turns on/off, and not the muscle active state because activation dynamics (relating electrical activity to cross bridge formation) are not explicitly included.

One way to account for the activation dynamics would be to incorporate them directly into the jump-diffusion model and numerically evaluate a solution. We chose a simpler approximation, and applied the activation dynamics on the muscle electrical activity 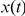 from Sanger's model to estimate muscle active state 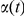. Activation dynamics was specified by the classic bilinear form [3]:
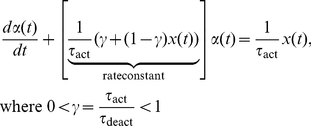
(3)


This differential equation models the history dependence in build-up of muscle activation, and captures differences between activation (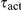) and deactivation (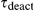) time constants with the ratio 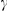. Notes on the biophysical relevance of our estimation procedure are available in Supplementary Text S2.

The muscle activation profiles estimated using our two-step procedure are shown in Figure 2. The intermediate Bayesian estimate 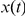 has a step-like shape as it primarily captures the turn on and turn off of the muscle electrical activity measured by EMG. The estimated activations 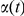 have profiles that are qualitatively expected from known temporal features of ankle muscle force build-up [4]. Further, the synergistic soleus and gastrocnemius muscles have similar profiles. Random step to step variations in EMG signals do not drastically change the estimated activation profiles. A repeatable ensemble average was obtained in as few as eight trials in cases of minimal motion artifact. The ensemble average estimates (Supplementary Text S2) show little variability in turn-on/turn-off times, and show greater variability in amplitude features (particularly when activation is high). The method and resulting estimates were found to be quite robust to normal, day-to-day variations in electrode placement for a given subject. We used our estimates of neurally stimulated muscle activations observed in walking to conduct the computational exploration (illustrated in Figure 1) of muscle-tendon morphologies.

### Mechanical and Metabolic Effects of Muscle Activations and MTU Morphologies

Using the muscle activations 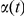 and joint kinematics 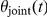 estimated from normal walking data, we actuated the leg muscle-tendon model 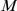 parameterized by a set of morphological features 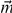. The parameter vector 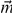 comprises the tendon reference strain 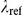, the tendon shape factor 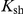, the muscle maximum isometric force 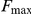 and the tendon slack length 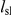 for each of the three ankle MTUs. We randomly generated sets of leg muscle-tendon parameter vectors, 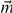 (from a uniform distribution with bounds stated in Supplementary Text S1), and computed both the model ankle torque profile, 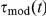 and metabolic energy consumed, 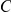, for each set:
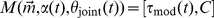
(4)


**Figure 3:**
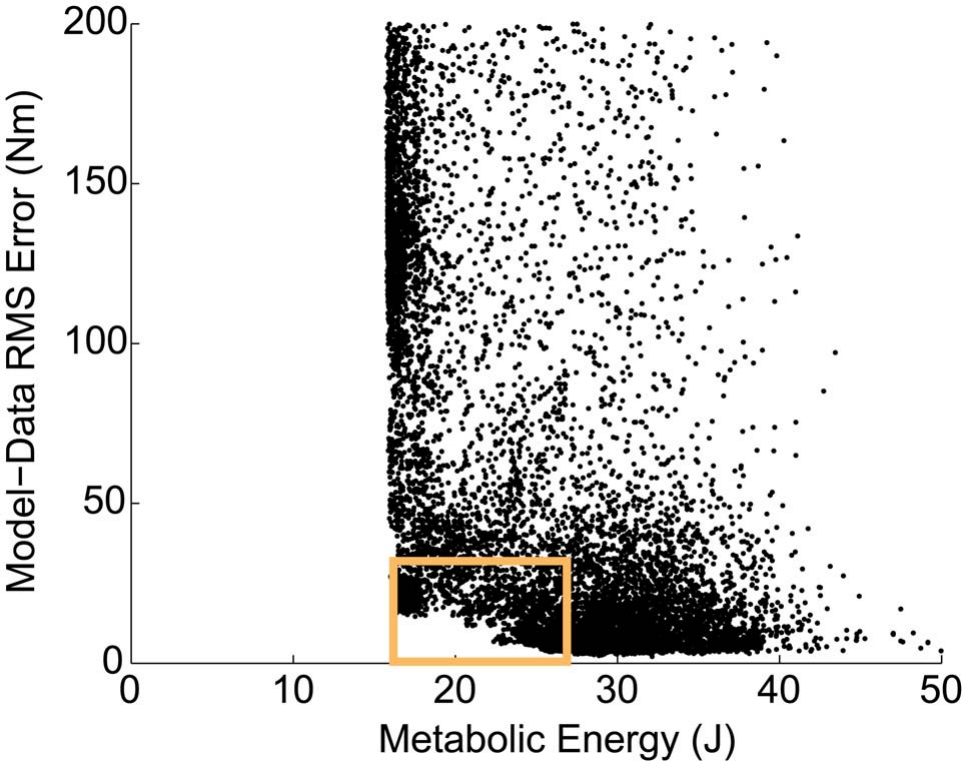
Relation between model dynamics and metabolics across the leg parameter space for normal gait data. Each point encodes a different parameter vector 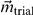, and therefore a different leg morphology. The torque axis is defined relative to human ankle torques in normal walking. The metabolic consumption values are calculated as outlined in Methods. The corner region, marked by the box, is the region corresponding to biologically realistic ankle torques and metabolic energies. The dimensions of the box correspond to the normal range (within error) of the ankle torques (from kinetic data obtained in this study) and metabolic energies (from independent studies [8], [9]). Points in the corner region have similar values for the morphological parameters defining them. Coefficients of variance indicating spread amongst parameter vectors in this corner region are within 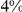 for most of the 12 parameters: 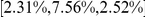 for the SOL, GAS and TA reference strains, 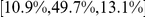 for the SOL, GAS and TA shape factors, 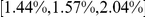 for the SOL, GAS and TA slack lengths and 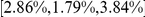 for the SOL, GAS and TA muscle isometric forces respectively (more details in Supplementary Text S3).

The resulting errors between model and human ankle torques are plotted against the model metabolic consumption (Figure 3).

Notable features of the plot include (a) the overall L shape, (b) a vertical boundary evidently representing the minimum energy that model muscles have to expend given the inputs, regardless of torque match and (c) an evidently systematic horizontal boundary below the population representing the best match between model and data. Each point along this horizontal boundary corresponds to a different metabolic consumption for the same level of error between model and human dynamics. A published empirical estimate of the range of metabolic consumption for ankle actuation in walking [8] is indicated, and is seen to be well-approximated by points exhibiting near-minimal economies, close to the the vertical boundary.

Remarkably, this overall parameter-space characterization reveals that the empirically observed realization is among the most economical of the many ways to produce human-like torque. Thus the human leg and the nervous system controlling it resolve the load-sharing redundancies in actuating the ankle most economically.

Points that best approximate human-like dynamics *and* optimal human-like metabolics lie near the bottom horizontal and left vertical boundary respectively. Thus points representing a logical intersection of the model's ability to best produce both human-like dynamics and metabolics lie in a small region at the lower-left corner (indicated by box in Figure 3). Points in this region not only have similar values of the torque and metabolic cost but also have similar values for the morphological parameters defining them. The coefficients of variance amongst parameter values in the corner region, listed in the caption of Figure 3, are low for most of the parameters (details in Supplementary Text S3). Further, all points outside the corner region compromise on either torque match, or economy, or both.

Thus, parameter vectors defining the corner region points can be identified computationally by encoding the simultaneous realization of two objectives (torque match and optimal economy) into a multi-objective problem. Solutions for such problems are generally sets of points that simultaneously realize both objectives as best as possible. These solutions, known as Pareto solutions, typically form a frontier along which the two objectives can be traded off against each other to varying degrees. In the special case that both objectives logically intersect at a mathematically sharp corner, there is a single strong Pareto optimal solution that best fulfils both objectives without any tradeoffs. As demonstrated above, our problem resembles this special case - within systematic limits of experimental precision, data variability and functional relevance. Thus it is possible to interpret our problem within the strong Pareto optimal framework, and simplify standard multi-objective optimization methods (such as Aggregate Objective Functions, Pareto ranking, evolutionary algorithms, or cost-constraint techniques [23]) to solve for the biologically realistic parameter vectors.

Our simplified approach relies on the observation that biologically realistic muscle-tendon morphological parameters (henceforth referred to as 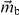) should (a) produce the normal human walking mechanics, *and* (b) minimize metabolic cost. To solve for 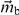 we take a two-step path: (a) restrict the search to parameter vectors that enable the model to produce human-like torque (horizontal boundary), and (b) look, within the restricted space, for parameter vectors that optimize economy. Thus, the problem of finding 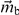 is akin to a constrained optimization, performed by generating candidate parameter vector populations and iteratively focussing the search on the biologically realistic left corner (Methods).

For each of the five subjects, we used the training gait data to obtain activation and joint state estimates, automated the above exploration to find corner region parameters (listed in Supplementary Text S3) and defined the model with the optimal vector to be the ‘trained model’. We cross-validated the trained model against variations in input data (Supplementary Text S3) and proceeded to characterize the biological relevance of the trained model morphology.

### Biologically Realistic Morphologies and Relevance to Metabolic Economy

The optimal leg morphological features 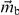 for each subject were seen to fall within physiological ranges [18]. To gain insight into non-apparent features underlying the solution, we extracted both functional and geometrical features that significantly influence muscle-tendon action and the associated metabolics: (a) tendon stiffnesses and (b) muscle-tendon rest length ratios.

To compute these metrics, the trained model dynamics were solved numerically (Methods) to obtain muscle lengths 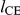, muscle velocities 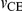, tendon lengths 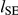, muscle-tendon unit force profiles 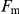 and model ankle torques 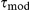. Tendon stiffness was approximated as the best fit slope of the tendon force-length relation defined by the computed morphologies 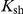, 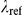 and 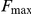 (Methods). Only regions of the tendon 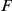
–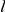 curve where force was over 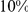 of the peak force were considered to prevent the non-linear toe regions from artificially reducing the stiffness estimates. Geometric metrics were computed using the optimized morphological features. Since the ankle metabolic cost guiding identification of biologically realistic morphological parameters is dominated by stance-phase activity of the powerful soleus and gastrocnemius muscles, we focus on predictions for these two plantar flexor muscles.

Table 1 highlights the MTU structure trends. Notably, the model soleus and gastrocnemius tendon stiffness values (kSOL and kGAS) are quite compliant and lie within literature ranges [12], [24]. While the stiffness trends encapsulate effects of parameters 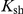, 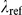 and 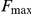, the effect of slack length 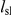 is captured in a geometric effect described in the last two rows of Table 1. The ratio of muscle rest length 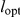 to the computed tendon slack length 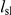 is conserved for both plantar flexor muscles across subjects. This trend is consistent with published human cadaver studies as well [25].

**Table 1:** Trends in optimal muscle-tendon morphological parameters.

Subject	1	2	3	4	5
**kSOL [N/mm]**	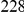	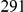	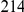	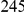	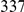
**kGAS [N/mm]**	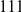	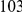	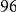	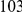	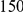
**SOL** 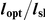	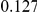	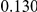	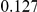	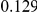	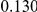
**GAS** 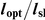	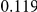	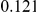	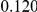	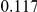	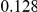

Next, we sought to understand the significance of the optimal morphologies (specifically as embodied in the above in tendon compliance and the conserved 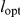/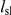 ratio trends) to metabolic economy. For this, we compared a metabolic efficiency metric accounting for the effects of tendon elasticity against the efficiency of muscle positive work alone. Muscle positive work efficiency was computed based on the metabolic cost of muscle mechanical work during active shortening (Equation 12). For comparison, a net joint level mechanical efficiency based on the total metabolic cost of performing mechanical work at the joint (inclusive of muscle work during active shortening, active lengthening and passive tendon contributions) was calculated (Equation 13). In the latter case, the metabolic and mechanical calculations are not restricted to muscle positive work phases, as the MTU dynamics can allow tendons to perform positive mechanical work at the joint even when the muscle cannot.

Table 2 details the resulting muscle positive work efficiency and the overall joint mechanical work efficiency. The average stance phase efficiency of muscles doing positive work (without regard to storage and release of elastic energy) is 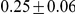. This is consistent with empirically measured performance of isolated skeletal muscle doing positive work [8], [16]. Though the plantar flexor muscles themselves perform at ordinary efficiencies, accounting for tendon elastic energy contributions boosts their efficiency in performing joint mechanical work to a high net ankle mechanical efficiency of 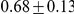 during stance phase (Table 2). To ensure this is not an over-estimate due to neglect of tendon viscosity, we recalculated with a nominal viscous loss of 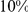 of the tendon elastic energy [18] - and obtained a 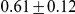 joint work efficiency, still 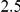 times higher than positive muscle work efficiency. The observation that accounting for elastic energy affords a dramatic increase in efficiency of joint work is qualitatively consistent with another recent report [8].

**Table 2:** Contribution of tendon elasticity to overall metabolic efficiency of joint work.

Subject	1	2	3	4	5
**Metabolic Cost for Positive Muscle Work (J)**	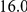	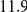	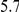	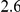	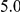
**Positive Muscle Mechanical Work (J)**	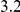	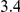	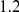	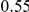	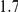
**Efficiency of Positive Muscle Work**	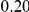	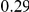	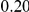	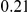	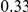
**Total Metabolic Cost (J)**	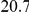	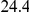	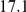	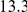	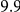
**Net Mechanical Work at Joint (J)**	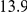	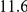	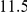	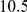	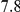
**Mechanical Efficiency at Joint**	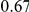	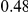	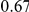	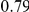	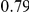

Efficiencies calculated with and without accounting for mechanical work contributions from the elastic tendon are compared

Thus the biologically realistic morphologies correspond to compliant tendons that store and release elastic energy to enhance joint work with little extra metabolic cost to muscles. As the elastic storage and release is timed to allow muscles to work efficiently, there is an optimal tendon slack length 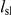 that is tuned to muscle optimal length 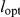 and the input activation profiles. In summary, our exploration of the muscle-tendon morphological space predicts that the optimal muscle-tendon morphologies enable the nervous system to drive ankle muscles in high performance regimes.

### Muscle and Tendon Operation within Plantar Flexor MTUs

**Figure 4:**
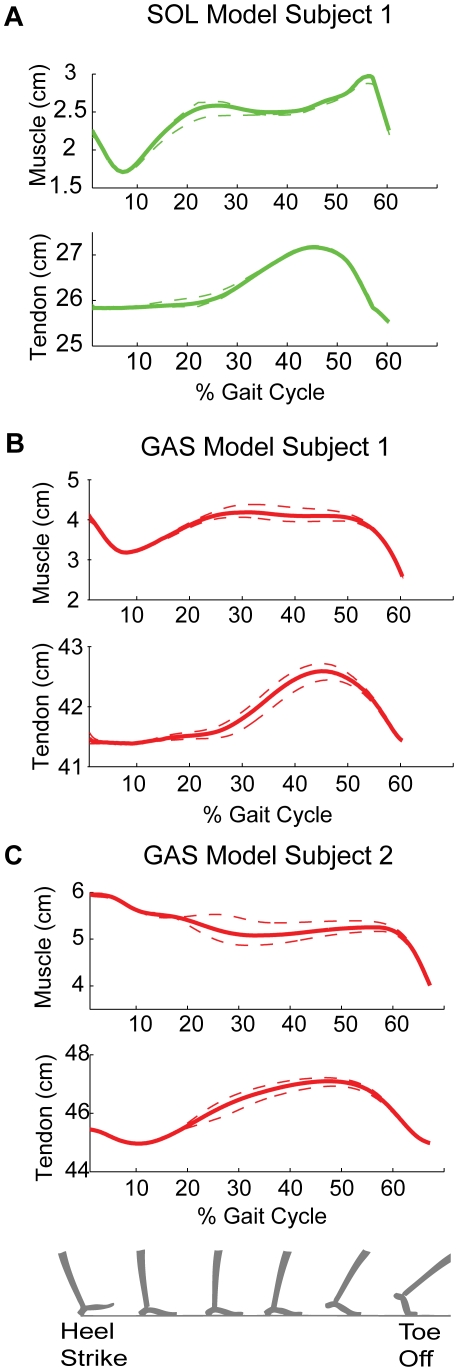
Model predictions of soleus and (medial) gastrocnemius muscle fascicle and tendon lengths. Panel A displays soleus muscle and tendon length predictions, while Panels B and C display gastrocnemius muscle and tendon length predictions for two different subjects. Dashed lines in panels A, B and C represents standard deviations, propagated from standard deviations of data-based activation and muscle-tendon unit length estimates. Notable features across the panels include (a) the relatively small changes in muscle lengths for much of stance, (b) the slow stretch of tendons in mid-stance, and (c) the rapid recoil of tendons in late stance. These observations are qualitatively consistent with previously published ultrasonography-based measures on walking humans [5], [6]. Model muscle-tendon unit lengths (not shown) are directly related to the model muscle lengths and tendon lengths. Soleus and gastrocnemius actions have distinct features. Soleus fascicle strain profile has an eccentric phase between 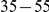
% GC, while gastrocnemius fascicle length is largely isometric between 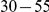
% GC. The gastrocnemius fascicle strain profile is variable across subjects during the early stance and toe-off phases of the gait cycle.

We queried the model for further details regarding individual muscle and tendon operation regimes. Plantar flexor muscle and tendon length estimates from the model are shown in Figure 4. Across subjects, both soleus and gastrocnemius muscle strains were noticeably less than tendon strains. Plantar flexor tendons are stretched slowly over most of stance, and released quickly before toe-off just as the muscles shorten rapidly. In accordance with observations in the previous section, we see that the optimal morphologies enable the timely storage and release of tendon elastic energy (stretching and shortening of tendons), giving rise to efficient (near-isometric) muscle operation. The model's plantar flexor muscle strain predictions are qualitatively consistent with trends reported in independent ultrasonography-based *in vivo* measures [5], [6].

Further, the model captures the diversity represented in the *in vivo* data from different studies. For the gastrocnemius muscle, model profiles (Figure 4, Panel B) are consistent with ultrasound recordings reported in [5] for some subjects, and with the measures from [6] for other subjects (Figure 4, Panel C). Specifically, there are differences in early stance action that appear to arise largely from differences in early stance ankle angle, and orientation of the foot at the moment of ground impact. There are also differences in the degree of peak shortening towards toe-off. Thus, our results suggest that qualitative trend variations among different *in vivo* measures [5], [6] may arise from subject-to-subject gait variations and not necessarily due to differences in the ultrasonography techniques.

Beyond these qualitative observations, model soleus muscle peak strains (Figure 4, Panel A) are quantitatively consistent with those published in [5]. But quantitative differences exist between model predictions for the gastrocnemius muscle (Figure 4, Panels B and C) and the two sets of *in vivo* measurements. Model gastrocnemius peak shortening strains range from 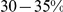, while [5] and [6] report peak shortening strains of 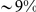 and 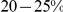 respectively. To understand the reason for these differences in muscle strains, we studied the tendon and MTU strain profiles. Interestingly, model tendon lengths (Figure 4) and MTU lengths (not displayed) agree quantitatively with both sets of *in vivo* measures. However, this does not translate to quantitative agreement between model and the *in vivo* muscle strains. Since muscle length is a geometric function of the tendon and MTU lengths, the quantitative differences can be attributed to inconsistencies between the model's geometry and the complex *in vivo* geometry. Sources for discrepancy include (a) dimensions of the subjects studied, and (b) differences between our lumped element model geometry and the true anatomical geometries, arising possibly from the two-dimensional nature of our analyses (no volume or shape considerations) and from other model simplifications like constant pennation angles. Nevertheless, the overall trends in model muscle and tendon strain predictions are robust to these errors, and empirically realistic.

**Figure 5:**
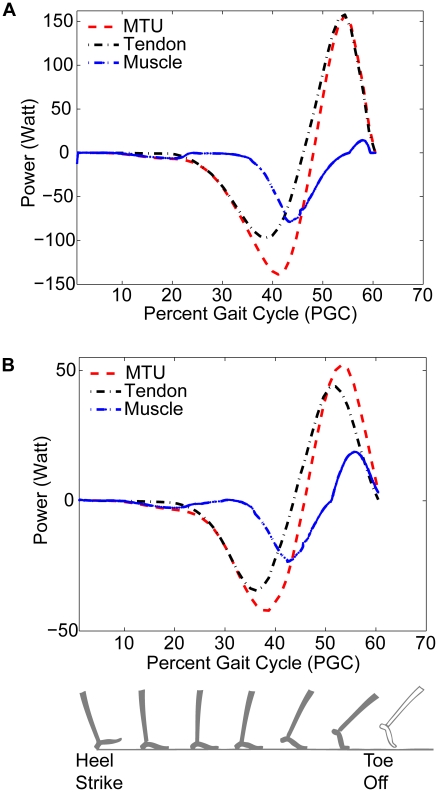
Breakdown of power generation within the soleus and gastrocnemius MTUs. Panel A shows model predictions of soleus muscle, tendon and MTU power, while Panel B shows similar predictions for the gastrocnemius group. Powers are computed along the tendon axis, and considered positive during shortening phases for each element (muscle, tendon and MTU). 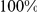 gait cycle is equivalent to a stridetime of 1.1 seconds for the gait cycle displayed. For both the MTUs, tendons contribute much more to the MTU power output than do muscles - especially during late stance. Soleus has higher peak MTU power than the gastrocnemius MTU primarily because the soleus has a larger cross-section area.

The value of our modeling effort extends well beyond enabling comparisons between our theory and published empirical measurements. Difficulties in directly measuring individual muscle and tendon forces within a muscle-tendon unit have precluded resolution of how the total MTU power output breaks down between the muscle and the tendon. Our analysis provides estimates of individual muscle and tendon forces, and therefore enables calculation of muscle power and tendon power within each MTU - as displayed in Figure 5. The most striking feature of these plots is that much of the MTU power arises from the tendons not the muscles. In particular, during the late stance positive power generation period, tendons provide over 80% of the MTU power across subjects. This is consistent with the above observations of tendon strains being much larger than muscle strains for both plantar flexors. Overall, the soleus MTU has higher peak MTU powers than the gastrocnemius MTU. This granularity of information motivates a more detailed study of similarities and differences in the operation of the different muscles and tendons.

### Roles of Plantar Flexors and Load-Sharing

The synergistic soleus and gastrocnemius muscles are similar in that they shorten significantly right before toe-off, and move with low velocities, as is expected from their compliant tendons. But there are two easily apparent differences in the movement of these two muscles - during mid and late stance respectively.

First, the length estimates of the two plantar flexors are very different in mid-stance (Figure 4). In particular, the soleus lengthens (eccentric operation) during mid-stance while the gastrocnemius appears characteristically isometric (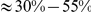 GC). Thus the soleus absorbs mechanical work in mid-stance, while the gastrocnemius holds the tendon in place at the muscle end and does little mechanical work. This observation is consistent with ultrasound literature reports [5], [7].

**Figure 6:**
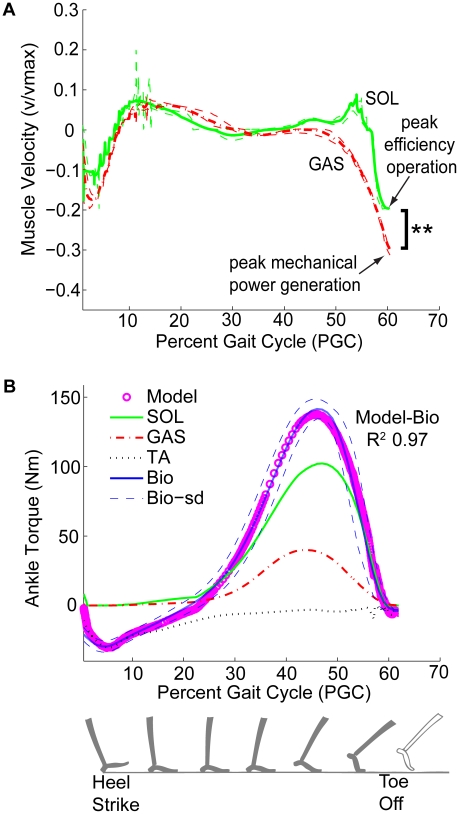
Roles and breakdown amongst different muscle-tendon units spanning the ankle. Panel A shows model predictions of soleus and gastrocnemius muscle (fascicle) velocity. Plantar flexor muscle velocities are close to zero through most of stance. Standard deviations (thin dashed lines) are obtained by propagating the standard deviations of the input activations and muscle-tendon lengths through the model. Soleus and gastrocnemius velocities are significantly different right around toe-off. The asterisks (**) indicate a paired difference 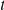-test significance with 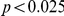. Panel B shows the breakdown of ankle torque amongst the different muscle-tendon units. Peak gastrocnemius torque is nearly half of the peak soleus torque. Total model torque is within normal variations of the biological ankle torques for this subject. Since it is only meaningful to evaluate muscle action when muscles are ‘on’ and working, figures are presented for stance phase only.

Moreover, there are differences in late stance operation of the two muscles, which are apparent from an analysis of muscle velocities (Figure 6, Panel A). During pre-toe-off shortening, the soleus operates at a peak velocity of 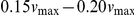, while the gastrocnemius operates at a larger peak velocity of 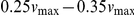 (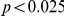). These peak toe-off velocities fall in well-recognized ranges. Muscle efficiency is known to peak around 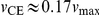 for a wide range of muscle lengths, while muscle mechanical power peaks around 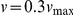 [13], [26]. Within precision of these empirical numbers, our results suggest that stance-end muscle operation may be driven by peak efficiency for the soleus and peak mechanical power for the gastrocnemius.

Motivated by this idea, we compared each muscle's positive mechanical work and metabolic consumption during the positive work phase of late stance. Table 3 reports ratios of positive mechanical work, metabolic energy cost and the resulting efficiencies of the two muscles in late stance. While the relation between soleus and gastrocnemius mechanical work and metabolic cost had varying trends across subjects, soleus is consistently more efficient than the gastrocnemius. In other words, soleus achieves a much bigger bang (mechanical work-wise) for its buck than the gastrocnemius. Further, the fact that the mechanical work ratios are low (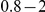) for most subjects - despite the fact that soleus is nearly 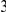 times as large (in cross-section area) as gastrocnemius - suggests that the gastrocnemius may be more powerful than soleus on a per fiber basis (due to the velocity difference noted above). The above results argue that soleus may be an economical force producing muscle, while gastrocnemius fibers may be more powerful and metabolically demanding than soleus fibers. Details of the metabolic and mechanical powers of the two muscles are available in Supplementary Figure S1.

**Table 3:** Efficiency of positive muscle mechanical work: Soleus vs. gastrocnemius.

Subject	1	2	3	4	5
**Ratio of Positive Muscle Work (SOL/GAS)**	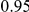	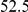	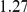	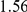	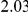
**Ratio of Metabolic Energy Consumed for Positive Muscle Work (SOL/GAS)**	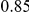	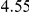	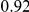	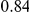	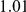
**Ratio of Positive Muscle Work Efficiencies (SOL/GAS)**	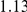	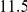	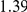	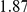	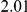

To further elucidate roles of the two plantar flexors, we studied the metabolically optimal breakdown of ankle torque between the two (Figure 6, Panel B). The ratio of peak soleus and gastrocnemius torque contributions to ankle actuation is an average of 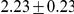 across subjects. This ratio does not directly follow either the ratios of the optimal 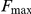 values or the metabolic costs of the two muscles. It is likely due to a combination of the 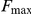, metabolic costs and the muscle activations. Interestingly, the most efficient partitioning of ankle torque amongst the synergistic plantar flexor muscles appears commensurate with the ratios of soleus and gastrocnemius stiffness reported in Table 1. This suggests that the soleus and gastrocnemius tendon extensions may be similar, which is just what we see in Figure 4.

Finally, the muscle operation and load-sharing results arise uniquely from the optimal parameter vectors. A point 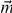 along the horizontal boundary of Figure 3 - that has a greater metabolic consumption than the biologically realistic corner points - also corresponds to (a) different muscle velocities than the optimal corner points (one-to-one relation between metabolic cost and muscle velocities), (b) different forces generated for the same activations, and (c) different (non-optimal) load-sharing solutions (see Supplementary Text S3).

## Discussion

Our results describe how humans resolve redundancies within and between MTUs involved in ankle joint actuation. We have demonstrated that there is a unique leg morphology which (a) most economically relates activations and angles from gait data with torques therein, (b) produces the above data via plantar flexor muscle motions, tendon motions, and metabolic performance that are consistent with experimental observations and (c) resolves empirically inaccessible features ranging from individual muscle forces and metabolic demand to mechanical power and working efficiencies. This morphology (defined by maximum isometric forces, tendon material properties and slack lengths for the ankle MTUs) is anatomically realistic, and Pareto optimal for the two objectives of torque match and efficiency.

To understand features of the morphology that enable this multi-objective optimality, we make a few observations about the solution. The optimal muscle isometric forces result in the most efficient breakdown of joint torque amongst the different MTUs. The tendon slack lengths balance the capacity for a timely buildup of force in response to the activations against the need to cycle tendon elastic energy for efficient force generation. Finally the optimal tendon material properties make for just the right stiffness values to produce the required joint torque but with just enough compliance to reduce muscle metabolic cost. These features indicate that leg muscles and tendons are designed to enable a metabolically optimal realization of human-like ankle mechanics under neural controls observed in normal walking.

Interestingly, the optimal parameters for any one MTU do not arise independently of those for the other MTUs, as both efficiency and torque match are net objectives for all the MTUs operating together. Rather the optimal structural parameters are a solution for the system as a whole to achieve the two objectives. Therefore, unlike the Lichtwark & Wilson study [12] that predicted the most efficient force generating design for an isolated MTU, our results predict the leg structure that most efficiently breaks down ankle torque amongst the different MTUs, and then the muscles and tendons within each MTU - all in an empirically consistent manner.

Further, the load-division implied by the optimal leg morphology also reveals a role division amongst the different muscles. The gastrocnemius muscle has a very compliant tendon - allowing it to work isometrically like a clutch in mid-stance, store energy in the tendon slowly and release it rapidly in late stance to produce high mechanical power on a per fiber basis - akin to a catapult (as proposed in [5]). The soleus muscle, on the other hand, has a stiffer tendon and larger maximum isometric force 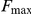 - making it very inefficient to generate high power by rapid shortening in late stance (as the metabolic cost increases commensurately with shortening velocities and scales with 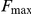). Thus soleus operates at lower muscle velocities in late stance, and can be thought of as an efficient force generator. To our knowledge, this is the first observation of differences in late stance operation of the two plantar flexor muscles in human walking, and remains to be tested in future experiments. Interestingly, previous studies have found differences in energy management by adjacent leg extensor muscles in insect locomotion [27], [28]. Thus differences in morphology of adjacent synergistically controlled MTUs may diversify MTU function across species. For smaller muscles like the tibialis anterior, which contribute little torque or mechanical power, the efficiency objective appears flat across the MTU design space. Nevertheless, these muscles may have important roles to play in fine control or sensing - that could be explored in a future study. Much of the biomechanics literature has focused on single objective problems. It has been acknowledged that multiple objectives could be acting in tandem [3]. Our results motivate the novel idea that one overall objective (of economically producing human-like torque) can give rise to different objectives (power, efficiency, control) for each individual element in the system.

It is possible that the neural controller may be ‘managing’ the different muscles and tendons spanning the ankle by ‘assigning’ different roles to each - based on their morphology - to efficiently accomplish ankle actuation in walking. In other words, the dynamical interplay between neural control (modeled here with estimated activations from human EMG data) and leg structure (modeled here with MTU morphologies) may in itself be optimal for the overall objective of efficiently generating ankle torque. This idea stands in contrast to previous proposals of the optimality of neural control alone [10], [11], [29] or of MTU structure alone [12] for a prescribed control or performance objective. The interaction between neural control and muscle-tendon unit mechanics may be facilitated by any subset of many neural pathways - particularly reflex pathways. However, there are many possible reflexes (muscle force [30], fascicle length and velocity [31], [32]) that can modulate impedance of ankle muscles at any given point in the gait cycle. This neural pathway redundancy has posed a challenge to decipher when and by how much each reflex pathway may contribute to activation of any given muscle. A systematic approach to quantify the role of specific reflexes and resolve this redundancy is desirable.

Our framework has implications as a starting point for such an endeavor. Since every reflex pathway is sensitive to specific changes in muscle state (force, length and velocity), inspecting the dominant trends in our muscle state and activation estimates provides insight on possible pathways contributing to the observed state changes. For instance, a period of muscle stretch and low activation followed by a period of isometric behavior and a coincident rise in activation is likely to correspond to a stretch reflex (gastrocnemius in mid-stance period of walking). A period of similarly shaped force and activation profiles may involve positive force feedback (soleus in late stance of walking). Such observations generate hypotheses on how impedance is modulated within the neuromuscular system. Forward dynamical simulations with perturbation analyses could used to test such hypotheses and quantify contributions of different reflexes to legged dynamics. Insights gained from such efforts are of interest for applications in the control of assistive devices [15]. Also, understanding the reflex responses that (along with tendon and MTU dynamics) modulate leg extensor muscle impedance after heel-strike may add perspective to studies on neuro-motor control during mechanical contact [33].

Finally, the framework described in this study also has implications as an analytical tool to probe empirically inaccessible metrics to understand regulation, roles, operation and performance of individual elements in gait. The first steps would be to extend the theory across muscles and joints for walking. Difficulties in obtaining inputs from gait and EMG data for deeper and more proximal muscles could be overcome via a forward dynamical simulation approach wherein both the timings of muscle activity, along with the muscle-tendon morphological parameters, are evaluated for our two objectives. If feedback control loops linking muscle state to activation are also included, perhaps other objectives of dynamical stability could be considered in tandem - in a similar framework, to quantitatively characterize the interplay between neural control and leg morphology. Accounting for feed-forward contributions to this interplay constitutes an important challenge that needs to be overcome. Another natural extension would be to characterize different tasks with our framework. A question of fundamental interest is to understand whether the same leg morphology is energetically optimal for the neural controls and joint mechanics across tasks. An affirmative answer would suggest that, for any specified task, humans select the joint mechanics that minimizes metabolic cost for the legs they have. A negative outcome would imply that human leg morphology and neuromuscular co-ordination are specifically energetically optimal for self-selected-speed walking.

## Methods

### Ethics Statement

This study was conducted in strict accordance with the principles expressed in the Declaration of Helsinki. The study was approved by the MIT Committee on the Use of Humans as Experimental Subjects (protocol number 0903003157). All subjects provided written informed consent for the collection of data, subsequent analysis and publication of results.

### Data

#### Data collection

Kinematic, kinetic and electromyographic (EMG) data were collected at an instrumented motion analysis facility in the MIT Computer Science and Artificial Intelligence Lab. Five healthy adult males participated in the study. After obtaining informed consent, participants were asked to walk barefoot at a self-selected speed (typically around 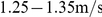). Walking trials within 5% of self-selected speed were accepted. For each participant, a total of 25–30 trials were collected. For two subjects, data were collected on multiple days (with consistent calibrations) to test robustness of the modeling and estimation techniques to day-to-day differences.

Standard procedures were used to collect the three types of data synchronously. Kinematic data was obtained using an infrared camera system (16 cameras, VICON motion analysis system, Oxford Metrics, Oxford, UK) to measure three-dimensional locations (precision 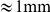) of reflective markers at 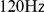. The markers, 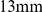 in diameter, were placed at 46 (bilateral) locations on the participant's body (Helen Hayes model) to track movements during trials. Kinetic data was collected using two back-to-back embedded platforms (Advanced Mechanical Technology, Inc., Watertown, MA) to measure ground reaction force and center of pressure locations (precisions 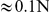 and 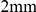 respectively) at 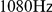. To ensure natural gait, subjects were not informed about the force-plate locations. Finally, surface EMGs were obtained using a 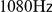 16 channel EMG system and MA-411 20X gain preamplifiers (Motion Lab Systems, Inc., Baton Rouge, LA); and disposable pre-gelled surface bipolar electrodes having 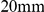 center-to-center spacing (Electrode Store Model BS-24SAF, part # DDN-20). Electrodes were placed on the soleus, medial gastrocnemius, lateral gastrocnemius and tibialis anterior muscles of one randomly chosen leg in the presence of a physician.

#### Obtaining joint motion, muscle-tendon geometries and joint dynamics

Raw marker and force-plate data were analyzed in SIMM (Software for Interactive Musculoskeletal Modeling, MusculoGraphics Inc., Evanston, IL) to obtain joint motion and dynamics. Using biomechanical properties in the SIMM Full Body Dynamic Model, inverse kinematics was performed to calculate joint angles, muscle-tendon lengths and moment arms [25]. Using the SIMM Dynamics Pipeline, inverse dynamics analyses were performed to determine joint torque profiles (arising from muscle-tendon contributions only, no external forces) and full body center-of-mass trajectories. All steady state walking data were split into gait-cycles and time-normalized to percent gait cycle (%GC) coordinates. Walking speed, stride length and timing of key gait cycle events were also calculated using the motion capture data.

#### Estimating muscle activation

EMG data was analyzed in MATLAB(R) (Mathworks, Natick, MA) to estimate the phase and amplitude of the underlying muscle active state. Raw EMG data for each muscle was pre-processed by removing DC offsets, clipping the signal amplitude to within five standard deviations, full-wave rectifying the clipped signal and then normalizing with respect to the peak value of the rectified signal [34]. The pre-processed EMG data was analyzed using the Bayesian algorithm [22], to estimate the neural excitation 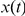 for each muscle. The bilinear excitation-activation dynamics (Equation 3) was solved in MATLAB Simulink to estimate muscle activation, 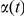. The activation and deactivation time constants governing Equation 3 were set to average values specified in [18]. The offset in the minimum estimated amplitude was removed to eliminate the noise-floor (when muscle was not on).

Plantar flexor muscle EMGs were occasionally corrupted by motion artifacts. The artifacts were prominent in the neural excitation estimates 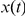 around the foot-flat period (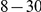
% GC for soleus, and 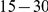
% GC for gastrocnemius). The artifacts were removed using a causal, 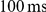 moving average filter applied on the neural excitation estimates right around foot-flat, while preserving the shape of the neural excitation profiles.

All gait data for a given subject were split into mutually exclusive training and testing sets. Within each set, ensemble averages and standard deviations of temporal profiles of each variable (joint angle, joint torque, muscle-tendon length, moment arms, and muscle active state) were used for analysis.

### Ankle Musculoskeletal Model

To investigate the leg dynamics underlying the data, we modeled the major muscle-tendon units contributing to ankle function in normal walking. Anatomically, this corresponds to the big MTUs responsible for ankle joint rotation in the sagittal plane - the soleus and gastrocnemius plantar flexors with the Achilles tendon split amongst them, and the tibialis anterior dorsiflexor (Figure 1). Both the medial and lateral heads of the gastrocnemius muscle were represented as one effective muscle, since they act synergistically in gait. Other muscle-tendon units spanning the ankle joint were not included as their contribution to ankle torques and energetics in normal, level-ground walking is minuscule [4]. The muscle-tendon dynamics actuating the ankle joint are outlined below.

#### Muscle dynamics

Each muscle was modeled as a unidirectional actuator with classic Hill-type dynamics, as in [30]. The muscle model consists of a contractile element (CE) representing active fascicles and a parallel elastic component (PE) representing connective tissue within the muscle. The contractile force 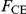 develops as a function of muscle active state 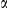, muscle fiber length 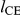 and contractile velocity 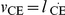. The parallel elastic element was modeled as a unidirectional non-linear spring, with force depending on 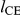. Muscle force resulting from both the contractile and parallel elastic elements is denoted by 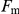. Muscle-specific parameters defining the dynamics include (a) the maximum isometric force 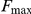; (b) the optimum fiber length 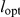 at which muscle provides the maximum isometric force, 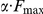 for activity level 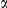; and (c) the maximum contractile velocity of the muscle 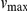 (mainly a function of the muscle's fiber composition).

#### Tendon dynamics

Each tendon is a non-linear elastic element in series with the corresponding muscle. Of the several approximations to tendon force-length relations in the literature, we chose a general non-linear form [16]:
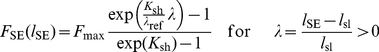
(5)where 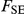, 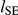 and 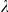 represent tendon force, tendon length and tendon strain with respect to slack length 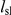 respectively. All parameters defining the tendon model 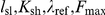 are morphological. 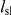 and 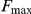 capture the dimensions, cross-sectional areas and space organization in the muscle-tendon unit. 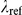 and 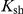 depend on the material properties and influence tendon stiffness. Parameter 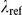 represents the reference strain at which 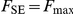. 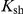 determines the shape and non-linearity of the length-tension curve.

#### Muscle-tendon unit dynamics

Each MTU comprises a muscle and a tendon connected in series, at pennation angle 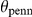 with each other. The MTU dynamics follows from the interaction between muscle and tendon, described by the first-order implicit nonlinear differential equation below:
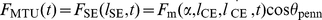
(6)

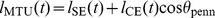
(7)where 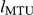, denoting MTU length, is related to joint angle 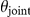 according to the leg geometry (as specified in the SIMM Dynamic Model). Each MTU has seven morphological parameters, three for the muscle dynamics, three for the tendon dynamics, and the pennation angle.

#### Joint dynamics

The overall ankle torque resulting from the three model muscle-tendon units was specified as:
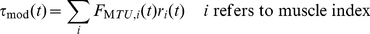
(8)where 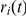 represents the time-varying moment arm for muscle 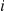 spanning the ankle. The moment arms were obtained from joint angles in the data using the musculoskeletal geometries in SIMM, as detailed in the Data section.

The full model dynamics was implemented in MATLAB Simulink, and is defined by 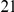 muscle-tendon morphological parameters, 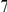 for each of the 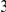 MTUs.

### Model Parameters, Inputs and Outputs

For each MTU, we minimized the number of free model parameters by (a) using literature values where they are known to be reliable (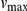 [30]), (b) fixing values in documented general ranges when dynamics are insensitive to precise values (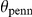 [35]), and (c) taking advantage of inter-relations between parameters (example, muscle optimal length 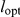 and tendon slack length 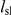 are inter-related by subject dimensions, so 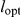 was set to a scaled nominal value from [35]). Set values for the above three parameters for each of the three MTUs are provided (with sensitivity notes) in Supplementary Text S1.

The other twelve parameters each correspond to a key morphological feature (slack length, reference strain and force, shape factor) of the three modeled muscle-tendon units. They are known to be difficult to measure *in vivo* [36], cadaver measurements are rather unreliable [25] and there is no fool-proof procedure for scaling nominal values from literature to subject dimensions [37]. Thus the model is characterized by twelve free parameters, denoted as 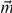.

The leg muscle-tendon unit dynamics, characterized by 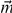, relates neurally commanded muscle activation (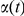) and joint angle 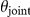 to joint torque 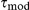. Considering a specified 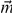, active state profiles for the three muscles and joint angles from the data (or equivalently the muscle-tendon unit lengths 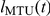) constrain Equations 5 and 6 for each unit. The relations can be simultaneously solved for 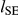 and 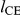 (implicitly 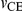), which in turn can be used to evaluate 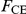 and 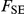 for each unit, and thus to calculate the model ankle torque 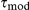.
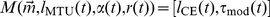
(9)


Each possible vector 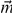 specifies a certain (a) model ankle torque, (b) distribution of that ankle torque amongst the MTUs, (c) division of mechanical work and MTU strain amongst the associated muscle and tendon. Since each parameter vector 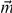 exacts different mechanical work from the model muscles, it also causes them to expend different amounts of metabolic energy to actuate the ankle.

### Muscle Metabolic Consumption

Muscle metabolic consumption is known to result from the heats of activation, maintenance, shortening, resting, and other molecular processes involved in muscle force generation [38]. While many schemes have been proposed to comprehensively account for these different components [10], [12], [39], they depend on setting several parameters correctly. To avoid accuracy and sensitivity issues that accompany multi-parameter metabolic calculations, we used empirically-based heat measures from classically accepted and well-reproduced muscle metabolic studies [13], [26], [38]. The data, reproduced in Supplementary Text S1 (along with sensitivity bands), relates the normalized metabolic power required for isolated muscle activity with the normalized contractile velocity.

At any time 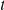, if muscle 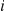 is activated to level 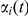, and is contracting at velocity 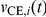, then the instantaneous (denormalized) metabolic power consumed by that muscle is:
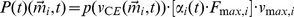
(10)where 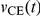 is implicitly a function of the parameters 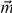 due to the specified muscle-tendon dynamics, 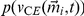 is the function in Supplementary Text S1, and the maximum isometric force when muscle 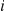 is activated to level 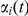 during a natural task is 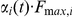. Overall metabolic energy cost for the muscle is the time integral of its instantaneous metabolic power:
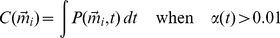
(11)


To avoid numerical errors in computing cost, metabolic power was only accounted for when 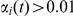, which is a small enough approximation that it does not affect accuracy. Total metabolic cost of actuating the ankle muscles in 1 gait cycle is 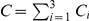 for the three muscles 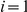 to 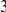.

In summary, given the data-driven inputs and model dynamics, each set of morphological parameters defining the dynamics specifies a model torque profile, and a model metabolic consumption - as indicated in equation 4.

For analysis, two efficiency metrics were calculated for stance phase of the gait cycle:

1. Muscle efficiency - accounting for positive mechanical work from the active muscle (during shortening) only:
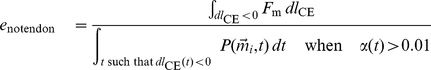
(12)
2. Muscle-tendon efficiency - accounting for both muscle and tendon contributions to the net ankle joint mechanics:
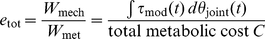
(13)

### Identifying Muscle-Tendon Parameters

#### Mathematical problem statement

Biologically realistic muscle-tendon parameters 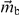 were identified via the constrained optimization procedure, motivated in the parameter exploration section of Results. Mathematically, the leg muscle-tendon morphology was specified as that which minimizes metabolic energy required to produce human-like dynamics:
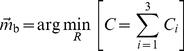
(14)where search is restricted to regions 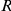 - in the space of all possible 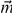 - satisfying the non-linear torque-match constraint:
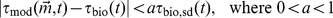
(15)



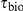
 is the ensemble mean biological ankle torque for a given subject, 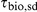 is the ensemble standard deviation in 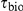 for the same subject, and 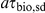 represents the narrowest band around the mean human ankle torque curve within which the model torque profile for that subject can lie. In other words 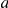 indicates the least RMS error between the model and data torques.

Cost is calculated for stance phase only, as swing phase metabolic consumption for ankle function is small and rather flat in the parameter space. Constraint was kept consistent with cost, and imposed point-wise during stance. As numerical errors in starting up the model and splitting up gait cycles make it difficult to satisfy the constraint of matching the steady state biological torque profile during 0–5% GC, the simulation was started at a point in the 0-5%GC range when the model-biological torque matching constraint was fulfilled and ran to completion of one gait cycle from there on out. Constraint violations between 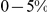 GC were discounted.

Identifying the most economical parameter vector for the best torque match gives the most energetically conservative estimates for 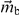 in the slightly rounded corner region of Figure 3. All analyses are reported for this most conservative point, even though the points in the corner region are similar in value and function.

#### Computational algorithm

The parameter space along the constrained landscape (horizontal boundary of Figure 3) was found to be rugged. Hence, a stochastic evolutionary method that could prevent entrapment in local minima was deemed appropriate. We chose the genetic algorithm (MATLAB Direct Search Toolbox) as it was easily integrated with the modeling and data analysis routines. The algorithm was implemented with settings that enabled speedy exploration of a diverse parameter space (Supplementary Text S1).

The non-linear constraint was enforced using a simple penalty method [40]. When the constraint was satisfied, the cost was just the metabolic energy. When the constraint was violated, a penalty proportional to the deviations between model and human torque was imposed:
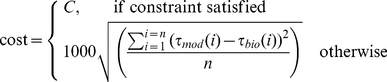
(16)where 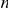 is the number of points during the stance phase. The penalty drove the optimization down the torque gradient into regions satisfying constraints. In the rare event that the penalty was steep enough to entrap the optimization in a particular feasible region, we used a population segregation approach [41] to diversify the search. Accuracy and robustness of the computational solutions to our problem were cross-checked by (a) inspecting the optimal parameter vectors for correspondence to features of Figure 3, (b) performing repeat runs with different starting points and follow-on gradient descent searches and (c) employing cross-validation checks against variations in inputs (Supplementary Text S3) as well as model assumptions (Supplementary Text S1) and (d) checking for biological features known from independent experiments (Results).

## Supporting Information

Figure S1 Plantar Flexor Muscle Metabolic Powers and Corresponding MTU Mechanical Powers. Panel A shows model predictions of soleus muscle mechanical power in relation to its tendon and MTU powers. Panel B shows model predictions of gastrocnemius muscle mechanical power in relation to its tendon and MTU powers. Mechanical powers are computed along the tendon axis, and considered positive during shortening phases for each element (muscle, tendon and MTU). Panel C shows metabolic power of soleus and gastrocnemius muscles. 100% gait cycle is equivalent to a stridetime of 1.16 seconds for the gait cycle displayed. Both muscles consume significant metabolic energy even when their MTU is doing negative mechanical work (∼15-50%GC). Comparisons between the mechanical and metabolic power trends for the two muscles in late stance are analyzed in Table 3.
(1.88 MB EPS)

Text S1 Model and Solver Settings. Model parameters motivated from literature, metabolic cost calculation details, bounds and algorithm settings used to identify optimal parameter vectors.
(0.45 MB PDF)

Text S2 Estimating Muscle Activation. Biophysical interpretation of the estimation procedure, along with estimated activation profiles for the 3 ankle muscles across 5 subjects.
(0.71 MB PDF)

Text S3 MTU Parameter Space Exploration. Supplementary notes on the biologically realistic points in the corner region, comparisons between corner region points and other points along the boundaries, tabulation of the optimal parameter values for 5 subjects.
(0.54 MB PDF)
