# Supplementary material for: Human Leg Model Predicts Ankle Muscle-Tendon Morphology, State, Roles and Energetics in Walking
Source: PLoS Comput Biol. 2011 Mar 17;7(3):e1001107. doi: 10.1371/journal.pcbi.1001107 (PMC3060164; doi:10.1371/journal.pcbi.1001107)
Supplement: Text S1 — Model and Solver Settings. Model parameters motivated from literature, metabolic cost calculation details, bounds and algorithm settings used to identify optimal parameter vectors. (0.45 MB PDF) [file pcbi.1001107.s002.pdf]

Model and Solver Settings

Fixed Muscle-Tendon Model Parameters

Table 1: Parameters defining muscle-tendon model dynamics

Parameter	Description	SOL	GAS	TA
$\mathbf{l}_{\text{opt}}$	muscle optimal length (meter)	0.030	0.045	0.098
$\mathbf{v}_{\text{max}}$	max. muscle contraction velocity (per sec)	$6l_{\text{opt}}$ $(5-8l_{\text{opt}})$	$12l_{\text{opt}}$ $(8-12l_{\text{opt}})$	$12l_{\text{opt}}$ $(8-12l_{\text{opt}})$
θ_{penn}	pennation angle (degrees)	25 (< 30)	17 (< 25)	5 (< 25)

l_{opt} was set based on scaled nominal values of all muscle-tendon parameters for an average adult male from literature [1]. Scaling algorithm from SIMM was followed. Since the Gastrocnemius muscle was modeled as an effective representation of the two heads, all parameters set were for the bigger, more pennate medial head. Maximal muscle contraction v_{max} values for all subjects were set as in [2], and the ranges indicated follow general trends in the literature. Modeling results hold as long as the soleus v_{max} is less than the gastrocnemius v_{max}. The parameter ranges indicate robustness of the modeling procedure and results to changes in set values.

Metabolic Cost Calculation

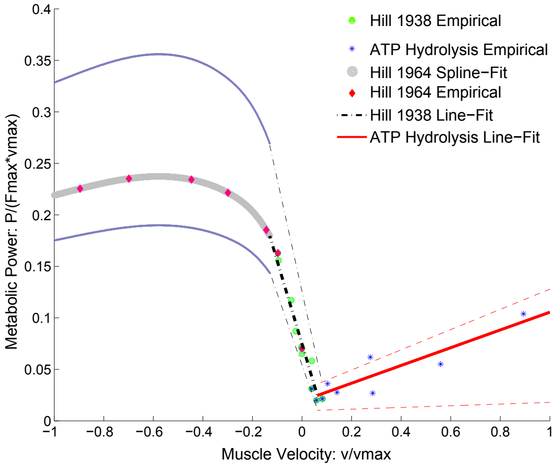

Figure 1: Empirically based muscle metabolic power as a function $p(v_{CE})$ of velocity (adapted from [3, 4]). Both power and velocity are normalized. Data for shortening are from Hill's original experiments and are widely accepted. Metabolic cost dominates during shortening. Data for lengthening are from ATP hydrolysis studies [3]. In general, empirical sources at high lengthening velocities are sparse but the common trends are accepted. Even though the data are collected under constant velocity contractions, they can be considered for dynamic tasks like gait because there is plenty of time (> 0.5 seconds) in between stimulation cycles for full recovery [4]. Trends in the figure are more important for our results than exact values. The dashed red, black and purple lines represent tolerances of qualitative trends in the optimization results to changes in the curve (such as changes that can occur from temperature variations, operating conditions or measurement accuracies).

Morphological Parameters Exploration

The search region was restricted to parameter regions within bounds motivated from nominal values based on subject dimensions and literature reports. The bounds for each parameter of the 3 ankle muscle-tendons (soleus, gastrocnemius and tibialis anterior) are shown in the table.

Table 2: Bounds for Muscle-Tendon Morphological Parameters

**Parameter**	**Description**	**Lower Bound**	**Upper Bound**
$F_{\mathbf{max}}$ **[N]**	Muscle Max. Isometric Force	0.5×nominal value	5×nominal value
$l_{\mathbf{sl}}$ **[cm]**	Tendon Slack Length	0.7×nominal value	$\frac{\min(l_{\mathrm{MTU}} - 0.5\, l_{\mathrm{opt}})}{\cos(\theta_{\mathrm{penn}})}$
$\lambda_{\mathbf{ref}}$	Tendon Reference Strain	0.02	0.09
$K_{\mathbf{sh}}$	Tendon Shape Factor	1	5

Bounds for muscle maximum isometric forces $F_{\max}$ and tendon slack lengths l_{sl} were based on nominal values derived from the data and subject dimensions using the SIMM Full Body Model. Nominal values were scaled by factors in ranges large enough to ensure feasibility for all subjects, regardless of height, weight and dimensions. In practise, while performing the searches, we ensured that the maximum soleus isometric force was larger than the maximum gastrocnemius isometric force. Further they were constrained to prevent muscles from generating force aphysically under slack. Absolute numerical bounds for the 8 λ_{ref} and K_{sh} parameters are motivated from literature reports [5]. As long as the $F_{\max}$ bounds allowed the soleus isometric force to take on larger values than that of the gastrocnemius, consistently with physiological expectations, the bounds did not influence the optimization results.

Multi-Objective Optimization Settings

Table 3: Genetic Algorithm Optimization Settings

**Option**	Population Size	Elite Count	Crossover Fraction f_c	Initialize	Vectorized
**Value**	150	5	0.8	Nil	On
**Value when Seeding**	150	2	0.4	Seed Value	On

Settings were chosen to optimize speed and diversity of the genetic algorithm in the MATLAB Direct Search Toolbox. Optimization runs were started from a random point (no seeding), unless seeding with a randomly chosen feasible value significantly speeded up convergence. Any decreases in population diversity arising from seeding were countered by reducing the cross over fraction, increasing the random mutation fraction, and decreasing elite count.

References

[1] Delp SL, Loan JP, Hoy MG, Zajac FE, Topp EL, et al. (1990) An interactive graphics-based model of the lower extremity to study orthopaedic surgical procedures. IEEE Transactions on Bio-Medical Engineering 37: 757–767.

[2] Geyer H, Herr H (2009) A muscle-reflex model that encodes principles of legged mechanics produces human walking dynamics and muscle activities. IEEE Transactions of Neural Systems and Rehabilitation Engineering .

[3] Ma SP, Zahalak GI (1991) A distribution-moment model of energetics in skeletal muscle. J Biomech 24: 21–35.

[4] Woledge RC, Curtin NA, Homsher E (1985) Energetic Aspects of Muscle Contraction. Academic Press.

[5] Authors Multiple Muscle Systems: Biomechanics and Movement Organization.
