# Supplementary material for: Human Leg Model Predicts Ankle Muscle-Tendon Morphology, State, Roles and Energetics in Walking
Source: PLoS Comput Biol. 2011 Mar 17;7(3):e1001107. doi: 10.1371/journal.pcbi.1001107 (PMC3060164; doi:10.1371/journal.pcbi.1001107)
Supplement: Text S2 — Estimating Muscle Activation. Biophysical interpretation of the estimation procedure, along with estimated activation profiles for the 3 ankle muscles across 5 subjects. (0.71 MB PDF) [file pcbi.1001107.s003.pdf]

Supplement: Estimating Muscle Activation

Biophysical Relevance of Estimation Procedure

In addition to capturing the phase and amplitude information in the EMG signal, the jump-diffusion model (wherein the jumps build up with a specified time dynamics) also captures some biophysical features of the muscle activation process itself. Neural stimuli can source or sink calcium ions from or into the sarcoplasmic reticula of a muscle. The calcium ions then set off a series of events to form cross-bridges and 'activate' the muscle. The cross-bridge formation process is history dependent and slow. The history dependence and rates are of biochemical origin, and are distinct for activation and deactivation (with the latter being slower)[1]. Thus jump processes, via a history dependent dynamics, drive a series of slow activation and deactivation events - as modeled in our estimation scheme.

The above reasoning indicates that the timing (turn on and turn off) of muscle electrical activity is the only critical feature extracted from the EMG data. Other aspects of muscle activity are calculated using the inherent biophysics of the build up of muscle activity or cross-bridge formation (manuscript Equation 3). If there were another way to obtain activity turn on and turn off times, an involved surface measure like EMG may not be required for estimating muscle activations.

Estimated Activation Profiles

The figure below shows activation profiles for all our 5 subjects. The dashed lines are ensemble average activation profiles, while the shaded areas represent the corresponding standard deviations.

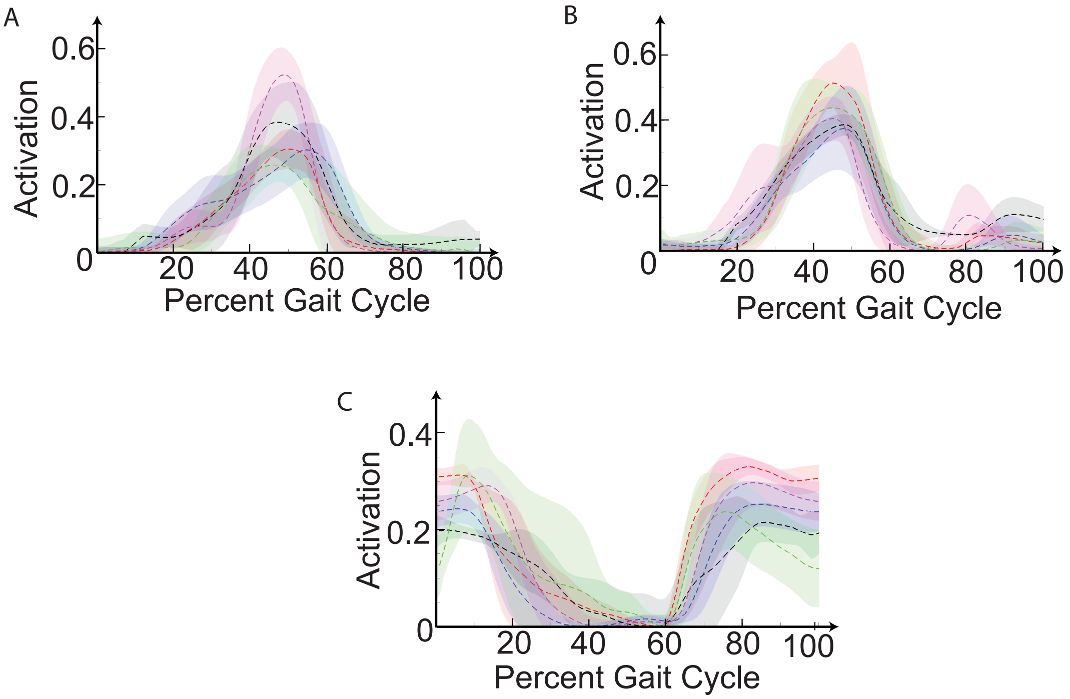

Figure 1: Activation estimates for 3 ankle muscles for 5 subjects (distinguished by color)

It is hard to directly validate our estimates as *in vivo* measurements of muscle activation are not available. An indirect validation may be performed on predictions from modeling schemes that use these activation estimates as inputs. Explanted muscle tissue experiments could also be designed to validate our estimation technique *ex vivo*.

References

[1] Hatze H (1977) A myocybernetic control model of skeletal muscle. Biological Cybernetics 25: 103–119.
