# Supplementary material for: Human Leg Model Predicts Ankle Muscle-Tendon Morphology, State, Roles and Energetics in Walking
Source: PLoS Comput Biol. 2011 Mar 17;7(3):e1001107. doi: 10.1371/journal.pcbi.1001107 (PMC3060164; doi:10.1371/journal.pcbi.1001107)
Supplement: Text S3 — MTU Parameter Space Exploration. Supplementary notes on the biologically realistic points in the corner region, comparisons between corner region points and other points along the boundaries, tabulation of the optimal parameter values for 5 subjects. (0.54 MB PDF) [file pcbi.1001107.s004.pdf]

Supplement: MTU Parameter Space Exploration

Trends in Morphological Parameter Value Space

The corner region points are defined by parameter vectors that have similar values. Low coefficients of variance (within 4%) were seen for most of the 12 parameters. Even though slight variations are observed in the parameters themselves (particularly the tendon material properties of reference strain and shape factor), the resulting tendon stiffness values are conserved across the parameter vectors in the corner region of manuscript Figure 3.

Further, the larger coefficients of variance come about from parameters taking on one of only a few values. For instance, the tendon shape factors K_{sh} defining the toe-region of the tendon force-length curve have coefficients of variance $> 10\%$ in the corner region, but each take on 1 of only 3 values in the region. Hence, although the tendon shape factors are not unique around the corner, they are not freely floating.

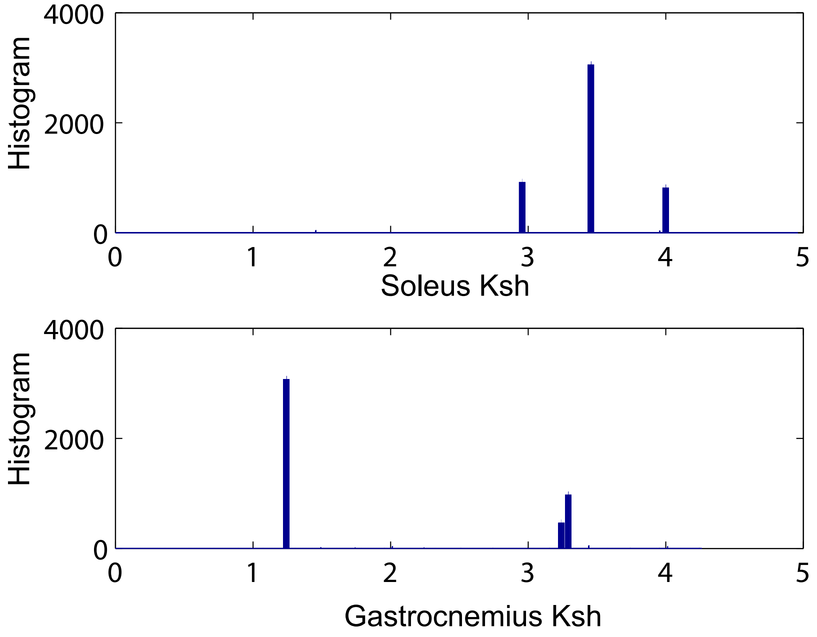

Figure 1: Variations in Corner Tendon Shape Factor Parameters for soleus and gastrocnemius.

Parameter vector values along the horizontal boundary vary considerably amongst each other and are different from the corner region parameter vector values too. The coefficients of variance along the horizontal boundary are $[33.1\%, 38.4\%, 11.8\%]$ for the SOL, GAS and TA reference strains, $[8.83\%, 50.1\%, 17.9\%]$ for the SOL, GAS and TA shape factors, $[1.21\%, 1.38\%, 2.98\%]$ for the SOL, GAS and TA slack lengths and $[8.61\%, 23.2\%, 22.9\%]$ for the SOL,GAS and TA muscle isometric forces respectively.

The slack lengths are the only parameters that have low coefficients of variance along the horizontal boundary, possibly to time the tendon engagement with the muscle activation profiles. The high coefficients of variance along the horizontal boundary shows that efficiency is an important factor for determining the biologically realistic parameters. Parameter vectors far from the corner along the horizontal boundary do not predict the biologically realistic morphologies and the many empirically observed features. In other words, the corner region is a unique predictor of the many empirical features.

Optimal Muscle-Tendon Parameter Values

Table 1: Optimal Muscle-Tendon Parameter Values

**Subject**	**1**	**2**	**3**	**4**	**5**
$\mathbf{F}_{\text{max, SOL}}$[**N**]	7142	5661	4520	4353	3974
$\mathbf{F}_{\text{max, GAS}}$[**N**]	1759	1847	1801	1786	1520
$\mathbf{F}_{\text{max, TA}}$[**N**]	2055	698	1243	1092	1245
$\lambda_{\text{ref, SOL}}$	0.083	0.073	0.082	0.086	0.080
$\lambda_{\text{ref, GAS}}$	0.042	0.060	0.062	0.037	0.066
$\lambda_{\text{ref, TA}}$	0.058	0.057	0.054	0.037	0.030
$\mathbf{K}_{\text{sh, SOL}}$	2.25	4.38	3.35	4.05	3.51
$\mathbf{K}_{\text{sh, GAS}}$	2.81	4.62	3.49	1.20	4.70
$\mathbf{K}_{\text{sh, TA}}$	1.12	1.90	2.14	3.92	4.00
$\mathbf{l}_{\text{sl, SOL}}$[**cm**]	25.8	27.9	25.8	26.5	21.8
$\mathbf{l}_{\text{sl, GAS}}$[**cm**]	41.4	44.9	40.1	43.9	34.0
$\mathbf{l}_{\text{sl, TA}}$[**cm**]	21.4	27.8	25.2	23.8	22.0
**Training Data** R^2	0.947	0.993	0.972	0.990	0.993
**Training RMS Error [Nm]**	8.34	4.47	7.30	3.71	2.81
**Testing Data** R^2	0.945	0.994	0.940	0.950	0.982
**Testing RMS Error [Nm]**	10.6	3.86	10.9	7.67	4.41

There is some repeatability in ranges of the parameters across subjects. The R^2 values and RMS errors provide statistical and physical metrics to quantify match between model and biological ankle torque. Average training data R^2 across subjects is a high 0.979, indicating efficacy of the automated procedure in converging to muscle-tendon parameter values that can explain the training gait data.

The trained model was cross-validated against variations in the input walking data. Estimates of angles and activations from testing gait data (independent of the training data used to identify corner region parameters) were used to quantify agreement between model torque τ_{mod} and testing ankle torques. Average testing data R^2 across subjects is also a high 0.962, indicating that the trained model has the dynamical range needed to capture normal trial-to-trial variations in the ankle data from human gait.

Match to knee torques was not explicitly enforced even though the gastrocnemius is a biarticular muscle, because a set of gastrocnemius muscle-tendon parameters matching the biological ankle torque can also match the late stance knee torque. This is because the gastrocnemius moment arm at the knee is nearly half its moment arm at the ankle. Studying the knee dynamics would require modeling all the thigh muscles and pose a computational burden with little to gain in terms of our modeling results. However, both knee and ankle joint kinematics were included in our evaluation of the biarticular gastrocnemius muscle-tendon length.
